# Consequences of toxic disasters for rescue, recovery, and clean-up workers require integrated mental and physical health monitoring: Editorial comment for social psychiatry and psychiatric epidemiology

**DOI:** 10.1007/s00127-015-1124-0

**Published:** 2015-09-19

**Authors:** Evelyn J. Bromet, Benjamin J. Luft

**Affiliations:** Stony Brook University, Stony Brook, New York; Edmund D. Pellegrino Professor of Medicine, Stony Brook University, Stony Brook, New York

## Introduction

Societies have an obligation to monitor and treat the health of workers participating in the clean-up of toxic disaster sites. Most of the research to date has focused on mortality and on mental or physical health, independent of one another. In this issue, Laidra et al. [1] present findings on the long-term well-being of Estonian men who assisted in the clean-up of the area around the Chernobyl nuclear power plant. Strikingly, 24 years later, these men, at an average age of 55, continued to have significantly more depressive and anxiety symptoms than controls, and were less likely to be employed and married and to describe their overall health as good. These findings complement and extend prior studies of Chernobyl clean-up workers that showed an increase in non-radiation related physical morbidity [2], PTSD, depression, and work absenteeism [3], and suicide [4]. Local Ukrainian studies have also raised the possibility of neurocognitive and brain-related impairments in highly exposed workers (reviewed in [5]).

Two other large-scale radiation disasters occurred in the last 75 years—the bombings of Hiroshima and Nagasaki in 1945 and the Fukushima nuclear power plant melt-downs in 2011. In contrast to numerous studies of radiation worker cohorts [6], we found no studies of the workers who handled the contaminated rubble in Japan. Similar to Chernobyl, the physical and mental health consequences of Fukushima are being monitored independent of one another. The physical health of clean-up workers is checked by government agencies, and mental health is being investigated in a separate research protocol [7].

Other large-scale toxic disasters have also occurred over the last 50+ years, including the Union Carbide gas leak in Bhopal, India, the sarin gas attack on the Tokyo subway, the oil spills in Alaska, Galicia (Spain) and the Gulf of Mexico, and the World Trade Center (WTC) disaster. Because such events occurred without warning and were protracted, medical monitoring and treatment programs and epidemiologic research were often established months to years later, after attention to the immediate impact of the disaster was rendered. For example, after the 2001 WTC disaster, the medical monitoring and treatment program for 9/11 responders funded by the Centers for Disease Control (CDC) was not initiated until July, 2002 [8].

There are multiple challenges inherent in developing strategies for understanding the long-term physical and mental health of clean-up workers after toxic disasters. In large part, these challenges stem from the uniqueness of these events with respect to the specific cultural, demographic and political contexts in which they occur, the varying degrees of physical, social and psychological trauma, and the varying levels of contamination that can make it impossible to establish individualized exposomes. All of these immediate factors interact with the workers’ personal disease predispositions. It is therefore essential to detail and characterize the nature of the exposure and its impact as close to the event as possible. This would require changes in funding mechanisms and a rapid response epidemiology corps ready to spring into action at the first sign of potential disaster. In contrast, to date most studies have been delayed and reductionistic, focusing on a specific disease or organ system, or mortality, leaving the extent and pathogenesis of multisystem disorders and symptoms poorly understood. This also hinders the design of multidisciplinary, maximally efficacious, intervention programs.

Another unique challenge for studies of clean-up workers after massive toxic disasters is the loss of trust in scientists. To a large extent, this is a consequence of the political issues surrounding responsibility for the cause and remediation of the disaster, graphic media reports on health effects that conflict with scientific findings, and a lack of experience among scientists in communicating science to the general public. How can studies of responders to toxic disasters be improved so as to enhance the translational value of the data?

## Some methodological challenges

The most critical challenges are developing and maintaining generalizable samples, implementing multisystem assessments with culturally relevant and internationally meaningful measures, and creating reliable exposure indices.

### Samples

Most samples of workers studied after toxic disasters are volunteers or individuals who self-enroll in monitoring programs [9]. There are exceptions, such as the WTC firefighters whose jobs require annual examinations and the Registry-based studies of Estonian clean-up workers [2, 4]. The limitations to generalizing from volunteer and monitoring samples are obvious [9]. But in the chaos following disasters, it is usually impossible to create a complete list of clean-up workers. The question then becomes: what are the benefits to translational science of studying volunteers and monitored samples? The answer lies in the nature of the questions being addressed. Obviously, these samples cannot address the incidence of disease. However, they can shed light on differential symptom and biomarker characteristics of exposed workers versus controls with the same diseases, disease remission and progression, and potentially unique comorbidity patterns associated with exposure levels.

Translationally useful research questions are often best addressed with longitudinal data. In this respect, monitoring program samples, especially programs with high retention rates, are immensely valuable. With appropriate consent, data collected routinely by the program can be combined with study materials. This is the case with the research programs on 9/11 responders monitored at the CDC’s WTC health programs.

### Multisystem, culturally relevant assessments

Disasters occur throughout the world, often in countries lacking a tradition of epidemiologic research. Baseline health status, life expectancy, and socioeconomic circumstances vary, and culturally specific idioms of distress differ widely. In many cultures, physical symptoms are the normative means for expressing distress. These issues have direct implications for conceptualizing and selecting the mental and physical health end-points and the selection of non-exposure risk factors.

The early studies conducted after Chernobyl primarily used local measures that did not translate conceptually to other settings. Later, collaborations with investigators from Europe and the US were undertaken, and internationally validated measures were implemented. Laidra et al. administered many such measures, including the Posttraumatic Stress Disorder Checklist (PCL), the SCL-90, and the Alcohol Use Disorders Identification Test (AUDIT), enabling comparisons with other cohorts. For example, Laidra et al. found that 38.9 % of Estonian clean-up workers scored ≥34 on the PCL. Applying the same cut-point to ~3500 WTC responders from the Stony Brook University WTC Health Program, we found a similar percent in this range among non-traditional responders (38.8 %) 11–13 years after 9/11. Laidra et al. also administered a local, validated measure of anxiety and depressive symptoms, thus optimally blending local and international measures.

As noted, most studies of clean-up workers have administered in-depth measures of mental health along with brief self-reports about physical health, or in-depth examinations of physical health with limited to no information on mental health, but not both. Given the multimorbidity of health and mental health [10], it is critical that studies of disaster workers who participated in the clean-up of toxic sites include state-of-the-art measures of both aspects of health. Given the time-consuming nature of such a comprehensive assessment, a 2-phase design, with physician administered diagnostic examinations limited to a targeted subsample of the cohort, is often optimal. This requires, however, highly trained staff and adequate and sustained funding, which is often lacking after massive toxic events.

### Exposure measures

Laidra et al. used registry information on year of arrival to approximate radiation exposure and found significant associations with insomnia, somatization, and anxiety. However, most toxic disasters involve multiple exposures that are not measured when the disaster occurs but are derived from questionnaires administered retrospectively. Such information decays with time and is subject to bias associated with current mood, anxiety, and health status. Since exposure severity is a critical risk factor that can also determine compensation, the development of valid and reliable measures and potential biomarkers of exposure are a priority for these studies.

## Conclusion

There has been a limited number of studies of disaster workers in general, and even fewer addressing the physical and mental health of workers exposed to high contamination sites, apart from the recent body of research on WTC responders. Because each toxic disaster is unique, replication studies are needed to identify consistent patterns of health effects that can be used for developing and tailoring intervention programs in the aftermath of these events. The translational value of multisystem, multi-symptom, long-term studies that shed light on the clinical pictures that ensue in the acute, intermediate, and long-term phases after toxic disasters is clear. The World Health Organization definition of health encompasses physical, mental, and social well-being. Future studies of disaster workers exposed to toxic elements will have optimal translational value by fully embracing all three features of the WHO definition of health.
